# *The Organon* and Materia Medica Club of the Bay Cities of California

**Published:** 1896-05

**Authors:** W. E. Ledyard

**Affiliations:** Secretary


### *THE ORGANON* AND MATERIA MEDICA CLUB OF THE BAY CITIES OF CALIFORNIA.

The regular semi-monthly meeting was held at the office of Dr. A. McNeil, 784 Van Ness Avenue, San Francisco, Friday evening, November 15th, 1895.

Members present: Drs. J. M. Selfridge, A. McNeil, S. E. Chapman, M. T. Wilson, W. E. Ledyard, G. J. Augur, M. F. Underwood, and George H. Martin.

The meeting was called to order at 8.10 o'clock by the President, Dr. J. M. Selfridge.

The minutes of the previous meeting were read by the Secretary, and after some corrections by Drs. Selfridge and Ledyard, were approved.

### DISCUSSION.

Dr. Chapman—I would like to ask Dr. Ledyard if he would remove the urine from the bladder by the use of a catheter if he were called to a case of uræmic poisoning? The urine in such a case acts as a poison to the system, and I have never been able to control a case unless I removed the urine.

Dr. Ledyard—I have not had occasion to use a catheter for fifteen years. The last time I did was in a case after confinement, and later I found out that if I had given a dose of *Arsenicum* it would have cured the case. I had a case of a little boy with horribly offensive stools; so bad that I hated to go into the house. The mother said that he had not passed urine for several days. *Apis*, from 200 to 20,000, would always pick that boy up from the verge of the grave.

Dr. Augur—I will relate a case to illustrate the fact that the indicated remedy, without mechanical means, will not always be sufficient. A young man eighteen years of age, a University graduate, was out with some friends. He sat down by a spring and took cold, which resulted in myelitis. A physician diagnosed it typhoid fever. When I saw him he could not pass water without the use of a catheter. I took a sponge wet with cold water and touched his back with it, and immediately he screamed out, "*It burns me—hot!*" There was entire paralysis of the bladder, and he was utterly unable to pass water. There was also a partial paralysis of the lower bowels, partial loss of sensibility in the extremities, as well as a partial loss of eyesight. I cured the case with *Opium*$^{200}$, occasionally repeated, but not often.

Dr. Ledyard—Did you draw the water with a catheter?

Dr. Augur—Certainly. Hammond says he cured but one case of myelitis.

Dr. Ledyard—Do you mean to say that in such a case the indicated remedy would never act without using the catheter?

Dr. Augur—It would act, but the boy would die, in the meantime, from uræmic poisoning if the urine was not removed.

Dr. Ledyard—So you positively assert, then, that the drawing off the urine cured, and not the indicated remedy?

Dr. Augur—I had to remove the urine to prevent shock from over-distention and to prevent the absorption of the urea, and then gave a remedy to cure the entire condition.

Dr. Ledyard—Why could not the *Opium* have cured without the catheter by allowing the urine to pass?

Dr. Selfridge—The bladder was extended to its fullest capacity. We know that if urea is retained in the system for twenty-four hours the patient will die.

Dr. Augur—It was three weeks before the boy was able to pass water naturally.

Dr. Ledyard—I know that *Opium* would have relieved him.

Dr. Augur—*Opium*$^{200}$ was the only remedy used.

Dr. Ledyard—But you drew off the water with the catheter and did not give the *Opium* an opportunity to do so.

Dr. Augur—How long do you suppose it would have been before he would have passed water voluntarily?

Dr. Ledyard—I do not know how long, but he would have done so.

Dr. McNeil read Note 3, of Section 7, of *The Organon.*

Dr. Selfridge—That covers it all, and I think physicians should use their judgment in all cases. When I was a medical student I knew of a case of a child in convulsions who was attended by Dr. S——. The child went a long time without a passage from the bowels. The doctor would not allow an enema, and though the people were good homœopaths, they soon became tired of this do-nothing business and called in another physician. He gave enemas and got away a foot and a half of fæcal matter. This fact did more good with these people than all Homœopathy might do.

Dr. Ledyard—That does not prove it at all to me. That physician may not have been a good homœopath, and may not have given the right remedy.

Dr. Martin—Does Dr. Ledyard ever use instruments in confinement cases?

Dr. Ledyard—Very seldom. I would use them, however, if I thought it necessary.

Dr. Chapman—Does Dr. Ledyard ever evacuate an abscess?

Dr. Ledyard—Not if I get the case early enough. I had a case of a boy with an abscess on the back of the neck. His people called it a boil, but it was not a boil. The neighbors wanted to treat it locally. It was a *Rhus* case. There was a large abscess at the nape of the neck, with a diameter of about three inches, rising up about an inch and a half. He came to me on Friday morning. I figured out about half a dozen or more symptoms and gave *Rhus*$^{200}$, one dose; and in twelve hours another powder to be dissolved if necessary, and to be given in from one to three doses. I said the abscess would break in thirty-six hours. On Sunday it did break, and the flow of pus kept up for half an hour. On Wednesday he came to me again, and there was not a vestige of the abscess left, with the exception of a slight line on the nape of the neck. If a poultice or knife had been used in this case the remedy would not have cured, because the action of the vital force would have been interfered with, it would have been opened too soon. The patient is feeling splendid, with a convalescent's appetite. This shows clearly the principle we have just been discussing.

Dr. McNeil—I have a case to illustrate this point. When I was a student my preceptor always made a crucial incision in abscess. When I began to read a little more for myself I was staggered by the statement that *Hepar-sulphur*$^{200}$ would cure abscess without the knife. A case soon came to me of a boy who had not slept for two nights from the pain of a boil. I sent him *Hepar-sulphur*$^{200}$. He went to sleep at ten, and the boil broke at twelve o'clock, and he was all right. Dr. Ledyard left out one point in his case. In my case the pain ceased very soon, but the abscess did not break for hours after.

Dr. Ledyard—As far as relief in my case was concerned the patient was relieved very soon.

The President then appointed Dr. Ledyard reader of the evening, who read Sections 1–8 of Dudgeon's translation of *The Organon.*

DISCUSSION.

Dr. McNeil—There is no danger of our ever getting ahead of Hahnemann, by the demands he makes upon us in the third paragraph; that is, to know the disease. Here is one point, that some pay no attention to pathology. To illustrate: say we are called into a case where there is vomiting, derangements of stomach; we naturally say, "What have we got here?" It may be cancer, ulceration, etc., etc., and as far as the remedy is concerned it does not make any difference what it is; but as far as the prognosis is concerned it does make a difference. When Hahnemann says the physician should know about drugs, he says that the physician should have an exhaustive knowledge of materia medica; and so he goes on through everything.

Dr. McNeil then read an article by Dr. G. M. Gould, of Philadelphia, and stated that the old school did not differ with us upon the question of vital force.

Dr. Chapman—Their antitoxines will lead them the right way if they keep it up.

The President then declared the meeting adjourned, to meet again the first Friday in December, at the office of Dr. J. M. Selfridge, in Oakland, when Wesselhœft's translation of *The Organon* would be read, as Dudgeon's translation was not liked by the members.

W. E. LEDYARD, M. D., *Secretary.*

Reported by Eleanor F. Martin, M. D.

---

